# Study Protocol: Phase III single-blinded fast-track pragmatic randomised controlled trial of a complex intervention for breathlessness in advanced disease

**DOI:** 10.1186/1745-6215-12-130

**Published:** 2011-05-20

**Authors:** Morag C Farquhar, A Toby Prevost, Paul McCrone, Irene J Higginson, Jennifer Gray, Barbara Brafman-Kennedy, Sara Booth

**Affiliations:** 1 General Practice and Primary Care Research Unit, Dept Public Health & Primary Care, University of Cambridge, Institute of Public Health, Robinson Way, Cambridge, CB2 0SR, UK; 2 Department of Primary Care and Public Health Sciences, King's College London, Captital House, 42 Weston Street, London, SE1 3QD, UK; 3 Institute of Psychiatry, Box P024, De Crespigny Park, London, SE5 8AF, UK; 4 Department of Palliative Care, Policy & Rehabilitation, King's College London, Cicely Saunders Institute, Bessemer Road, London, SE5 9PJ, UK; 5 Palliative Care Team, Box 63, Addenbrooke's Hospital, Cambridge University Hospitals' NHS Foundation Trust, Hills Road, Cambridge, CB2 0QQ, UK

## Abstract

**Background:**

Breathlessness in advanced disease causes significant distress to patients and carers and presents management challenges to health care professionals. The Breathlessness Intervention Service (BIS) seeks to improve the care of breathless patients with advanced disease (regardless of cause) through the use of evidence-based practice and working with other healthcare providers. BIS delivers a complex intervention (of non-pharmacological and pharmacological treatments) via a multi-professional team. BIS is being continuously developed and its impact evaluated using the MRC's framework for complex interventions (PreClinical, Phase I and Phase II completed). This paper presents the protocol for Phase III.

**Methods/Design:**

Phase III comprises a pragmatic, fast-track, single-blind randomised controlled trial of BIS versus standard care. Due to differing disease trajectories, the service uses two broad service models: one for patients with malignant disease (intervention delivered over two weeks) and one for patients with non-malignant disease (intervention delivered over four weeks). The Phase III trial therefore consists of two sub-protocols: one for patients with malignant conditions (four week protocol) and one for patients with non-malignant conditions (eight week protocol). Mixed method interviews are conducted with patients and their lay carers at three to five measurement points depending on randomisation and sub-protocol. Qualitative interviews are conducted with referring and non-referring health care professionals (malignant disease protocol only). The primary outcome measure is 'patient distress due to breathlessness' measured on a numerical rating scale (0-10). The trial includes economic evaluation. Analysis will be on an intention to treat basis.

**Discussion:**

This is the first evaluation of a breathlessness intervention for advanced disease to have followed the MRC framework and one of the first palliative care trials to use fast track methodology and single-blinding. The results will provide evidence of the clinical and cost-effectiveness of the service, informing its longer term development and implementation of the model in other centres nationally and internationally. It adds to methodological developments in palliative care research where complex interventions are common but evidence sparse.

**Trial Registration:**

ClinicalTrials.gov: NCT00678405

ISRCTN: ISRCTN04119516

## Background

Intractable breathlessness can completely dominate a patient's life causing physical disability, loss of independence and dignity and lowered self-esteem [1-3]. It is common in advanced disease, both malignant and non-malignant. In chronic obstructive pulmonary disease (COPD) and heart failure it is nearly universal by the time of death, its prevalence reaching over 50% in both conditions in the advanced stages. It occurs in 49% of the general population with all cancers [4], and in 90% of those with lung cancer [5]; these figures will rise as deaths from mesothelioma peak in 2016 [6]. The incidence of breathlessness in cancer is second only to that of pain [7]. Breathlessness is also a devastating accompaniment in less common cardio-respiratory diseases, e.g. interstitial lung disease and cystic fibrosis. Despite its prevalence breathlessness remains a poorly controlled symptom in which traditional pharmacological interventions are frequently ineffective [1,8,9].

However, patterns of breathlessness in cancer and COPD differ. Once patients with cancer become breathless they usually have a very short time to live and may become breathless at rest, whereas patients with COPD may live with gradually worsening symptoms and increasingly devastating consequences for years. As a result, patients with different diagnoses and their carers may have different needs [10]. The effects on the patient and family with malignant or non-malignant disease include increased social isolation, reduced activity, chronic anxiety, loss of employment and other changes in roles and perceived status [3,11]. Further, the economic burden to the healthcare system is significant [12].

Advances in the palliation of breathlessness include non-pharmacological intervention services to reduce or contain the severity of the sensation and, for patients with COPD, pulmonary rehabilitation programmes [13,14]. Non pharmacological methods for the management of breathlessness acknowledge the cognitive and emotional component [9]. Breathlessness services developed for patients with lung cancer have been formally evaluated with positive outcomes in terms of distress due to breathlessness, functioning and quality of life [15-17] however each of these studies had limitations: all were clinic based (patients were not seen at home); two were randomised controlled trials but one lacked published power calculations; and the outcomes reported by all were limited to patient outcomes i.e. none reported staff satisfaction, the views of referrers, or carer outcomes. Breathlessness studies have identified significant suffering among carers who report severe anxiety and helplessness as they witness their partners' suffering and feel powerless to reduce it [3].

In November 2003, a pilot Breathlessness Intervention Service (BIS) was set up at Addenbrooke's Hospital consisting of a clinical specialist physiotherapist and palliative care consultant [18]. BIS aims to manage the symptom of breathlessness in patients with any disease (malignant or non-malignant), using a rehabilitative approach. It uses a 'toolkit' of interventions including: evidence-based non-pharmacological interventions (psychological, social and physical); palliative care input (e.g. end of life issues, psychosocial issues, family concerns); and pharmacological review. Thus BIS seeks to enhance the self-management of breathlessness. Uniquely, care is located flexibly in clinic or in patients' own homes, as appropriate. Referrals come from medical specialists, GPs and allied health professionals (with medical consent).

From the outset its development and evaluation was grounded in the MRC's framework for the development and evaluation of complex interventions [19]. Following the developmental work conducted over the past twelve years (Pre-clinical-Phase II [3,10,18,20,21]), BIS is now undergoing evaluation in a Phase III definitive randomised controlled trial (RCT). This paper presents the Phase III RCT protocol.

The aim of Phase III is to address the following research questions:

1. Is BIS more effective than standard care for patients with intractable breathlessness from advanced malignant or non-malignant disease?

2. Does it reduce patient and carer distress due to breathlessness, and increase patients' sense of mastery of the symptom?

3. What are the experiences and views of those who use BIS, their informal carers and the clinicians who refer to it?

4. Is BIS cost-effective?

## Methods/Design

### Design

Phase III is a mixed method pragmatic fast track single blinded randomised controlled trial of BIS versus standard care.

**Figure 1:**
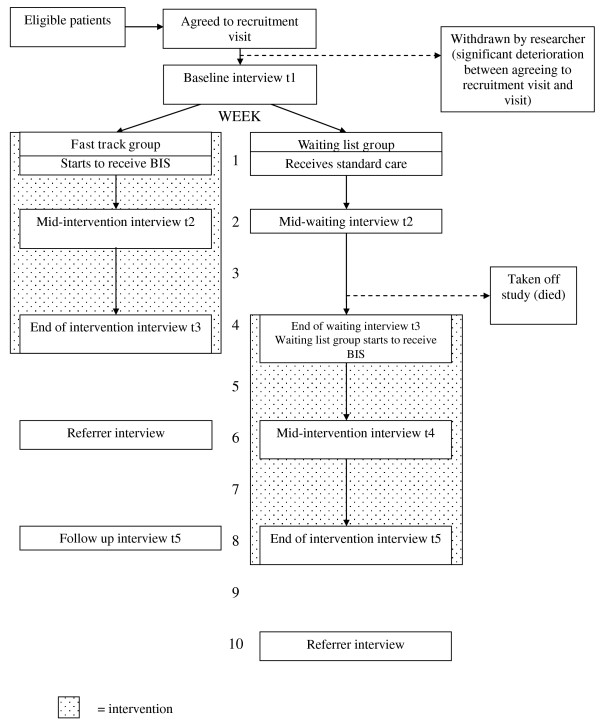
**BIS Phase III measurement point flow chart for non-malignant conditions**. shaded area = intervention

**Figure 2:**
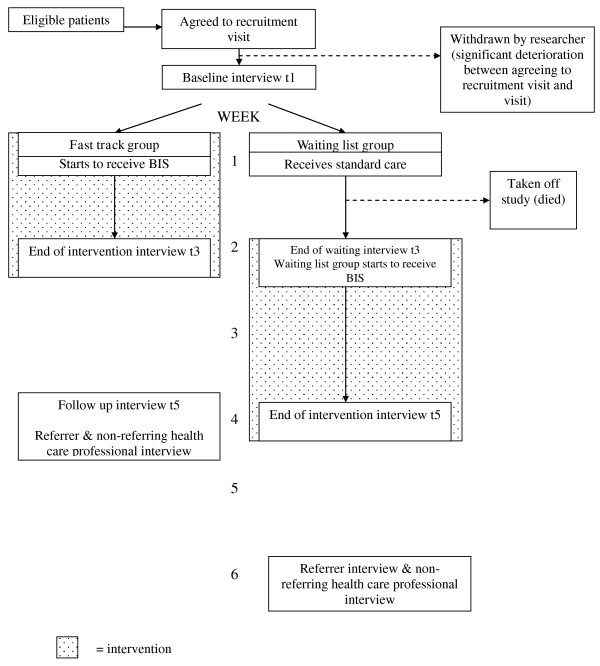
**BIS Phase III measurement point flow chart - malignant conditions**. shaded area = intervention

BIS offers care and support to patients with malignant or non-malignant conditions. Due to the differing disease trajectories of these two groups, BIS uses two broad service models: one for patients with malignancies (intervention over a two week period) and one for patients with non-malignant conditions (intervention over a four week period), summarised in Table 1. The trial therefore consists of two sub-protocols: one for patients with malignancies (four week protocol) and one for patients with non-malignant conditions (eight week protocol) (Figures 1 and 2).

**Table 1:** Service model for the Breathlessness Intervention Service (BIS) at Addenbrooke's Hospital, Cambridge University Hospitals NHS Foundation Trust (March 2008)

Disease group course:	Non-malignant (nm) course	Malignant (m) course
Examples:	e.g. COPD, heart failure, neurological disorders	e.g. all cancers, UIP

Referral:	Post, fax, electronic	Post, fax, electronic

Assessment lead:	Clinical Specialist Physiotherapist or Clinical Specialist Occupational Therapist	Medical Consultant (i.e. sees a doctor earlier in the intervention than non-malignant patients)

BIS team:	Clinical Specialist Occupational Therapist Clinical Specialist Physiotherapist Medical Consultant	Medical Consultant Clinical Specialist Occupational Therapist Clinical Specialist Physiotherapist

Medical assessment:	May be required	Always required (review of pharmacological management at 1^st ^visit)

First appointment:	Maximum wait of 3 weeks for first appointment	Maximum wait of 1 week for first appointment

Range of face-to-face visits:	2-3	1 (with primary care professional)

Average no. of telephone contacts (with patient/primary care staff):	3	2

Ratio of face-to-face to telephone:	1:1	2:1

Average length of service contact:	4 weeks + 16 week follow up (from 1^st ^assessment) post any pulmonary rehab/other referral	2 weeks + 6 week follow up (from 1^st ^assessment) post any pulmonary rehab/other referral

Service outcome measures collected at first assessment:	• modified BORG at rest, self-reported, on exertion and on completion of exercise test • anxiety due to breathlessness at rest, self reported, on exertion and on completion of exercise test • physiological measures e.g. oxygen saturation, heart rate	• modified BORG at rest, self-reported, on exertion and on completion of exercise test • anxiety due to breathlessness at rest, self reported, on exertion and on completion of exercise test (if acceptable) • physiological measures e.g. oxygen saturation, heart rate

Non-pharmacological interventions:	1^st ^stage of intervention	More likely to be concurrent with pharmacological interventions

Pharmacological interventions:	2^nd ^stage of intervention	More likely to be concurrent with non-pharmacological interventions

1^st ^stage interventions (selection & application as clinically indicated):	The majority of these interventions are used with this group, & taught over a longer period of time: → explanation & reassurance → anxiety management → psychological support → hand-held fan → information fact sheets → emergency plan → positioning to reduce work of breathing (rest, recovery & activity) → breathing control → education to patient, carer & health care generalists → pacing & lifestyle adjustment → individualised exercise plan → relaxation & visualisation → airway clearance techniques → advice regarding nutrition & hydration → support to family & patient to utilise education & self-support programmes → sleep hygiene → smoking cessation prompt → brief cognitive therapy → pharmacological review → well-being intervention → hypnosis → mindfulness CD → referral to specialist services (see below)	More selective use & application of these interventions, & taught over a shorter period of time: → explanation & reassurance → anxiety management → psychological support → hand-held fan → information fact sheets → emergency plan → positioning to reduce work of breathing (rest, recovery & activity) → breathing control → education to patient, carer & health care generalists → pacing & lifestyle adjustment → individualised exercise plan → relaxation & visualisation → airway clearance techniques → advice regarding nutrition & hydration → support to family & patient to utilise education & self-support programmes → sleep hygiene → brief cognitive therapy → pharmacological review → well-being intervention → hypnosis → mindfulness CD → referral to specialist services (see below)

2^nd ^stage interventions:	Choice of 2^nd ^stage interventions dependent on outcome of first stage interventions: → further pharmacological review e.g. low dose opioids, anti-depressants, anxiolytics → referral to specialist services (see below) → referral for LTOT or SBOT assessment	2^nd ^stage interventions likely to be applied concurrently with 1^st ^stage interventions: →further pharmacological review e.g. low dose opioids, anti-depressants, anxiolytics →referral to specialist services (see below) →referral for LTOT or SBOT assessment

Other symptom management:	May be required	Frequently required

Documentation:	→ individualised patient plan → summary to patient of outpatient consultation with medical consultant → discharge summary to referrer with copies to GP, specialist services the patient was already in contact with (e.g. respiratory physicians), other involved health care professionals (e.g. district nurses, nursing home care staff)	→ individualised patient plan → summary to patient of outpatient consultation with medical consultant →discharge summary to referrer with copies to GP, specialist services the patient was already in contact with (e.g. respiratory physicians), other involved health care professionals (e.g. district nurses, nursing home care staff) → supplementary medical letters more common

Referrals:	→ Pulmonary rehabilitation → Specialist dietetic → Specialist psychological services → Hospice day services → other specialist assessment → Cardiac rehabilitation → other rehab services (n.b. these services usually have a wait time)	→ Palliative care specialist service (n.b. rapid access available) → Specialist psychological services → Hospice day services → other specialist assessment

While understanding that it is not possible or desirable to standardize all aspects of the Breathlessness Intervention Service (BIS) for individual patients, we have established a service model with a minimum set of core interventions. The norm is for consultations to occur in the patient's own home (with clinically reasoned variance)

LTOT = long term oxygen therapy

SBOT = short burst oxygen therapy

Based on methods piloted at Phase II [20,21], consented patients are randomised either to a 'fast-track' group where they receive BIS immediately or to a waiting list group (control condition) where they receive the intervention after a defined period on a waiting list during which time they receive standard care. The advantage of a fast-track design is that it has the strength of a RCT, but with greater acceptability as all patients receive the intervention. Due to the palliative status of the patients referred to BIS, and the existence of BIS prior to this proposed RCT, a traditional intervention and control group design (where the control group would be denied access to the service) would be unethical and unacceptable [22]. This alternative fast-track design proved highly acceptable at Phase II [20].

### Setting

BIS is a secondary care service which provides care in a community setting, predominantly seeing patients in their own homes. It principally takes referrals from the Addenbrooke's catchment area seeing patients from Cambridgeshire, Hertfordshire and Essex and, where practical, further afield.

### Intervention

Table 1 outlines the minimum core interventions delivered by BIS. The service provides a thorough psychological and physical assessment taking into account patient and carer needs and breathlessness triggers. A treatment plan is agreed and implemented incorporating a range of evidence-based non-pharmacological and pharmacological techniques relevant to the patient and their lifestyle, helping them to self-manage their symptoms. A personal emergency plan is agreed and practised, with each patient receiving a copy. Paper copies of quality controlled information leaflets are provided (also accessible via the internet).

### Standard care

For the purposes of this trial, 'standard care' is defined as specialist outpatient appointments in secondary care (e.g. respiratory, cardiology, neurology or oncology) which may include specialist nurse input, and primary care services.

### Sample

The clinical team screen all referrals to the service against the study inclusion and exclusion criteria (Table 2) and a screening log is maintained.

**Table 2:** Inclusion and exclusion criteria

Inclusion criteria	Exclusion criteria:
Patients:	Patients/Carers:
(i) appropriate referral to BIS	(i) unable to give informed consent;
(ii) aged 18 years+	(ii) previously used BIS;
(iii) any patient not meeting any exclusion criteria	(iii) demented/confused;
	(iv) learning difficulties;
	(vi) other vulnerable groups e.g. head injury, severe trauma, mental illness;
	(vii) not meeting all inclusion criteria.
Carer:	
(i) informal carers (significant others, relatives, friends or neighbours) of Phase III RCT recruits	
(ii) aged 18 years+	
(iii) any carer not meeting any exclusion criteria.	

### Recruitment

A Patient Information Sheet is sent by first class post to eligible patients inviting participation in the trial. On the sub-protocol for patients with non-malignant conditions a second mailing is sent if there is no response within a week. On the sub-protocol for patients with malignancies a BIS clinician conducts a telephone follow-up if there is no response within a week; this clarifies that the patient has received a recruitment pack and establishes whether or not they would like to participate in the trial. If the patient (from either sub-protocol) refuses trial participation, or there is still no response, the service arranges to see the patient in the usual way and they are not recruited to the trial. If the patient responds positively to a recruitment letter a researcher contacts them to arrange to visit and explain the study in more detail, answer any questions, obtain informed consent, conduct the baseline interview (t1) and identify their lay carer.

If their lay carer is present at the patient's t1 then they are directly invited to participate in the study. If they are willing then the researcher answers any questions, obtains informed consent and conducts the carer baseline interview (t1). Where possible, carers are interviewed separately from patients as this was found to be important in the Phase I of the evaluation [3]. If the lay carer is not present then a recruitment pack (similar to that for the patient) is either left for them with the patient, or posted to them, depending on circumstances. They are then approached in the same way as for patients.

### Randomisation

Randomisation is carried out within 24 hours of the baseline interview (t1) by a third party (Addenbrooke's clinical trials' team) sequentially opening sealed opaque envelopes containing the random group allocation previously generated using a computer programme by the study statistician (University of Cambridge). The envelopes were set up by an administrator at University of Cambridge. Patients are allocated to the fast track group or waiting list group with an equal 1:1 allocation ratio using the method of stratified randomisation, with disease group (malignant, non-malignant) as the single stratifier.

Patients are informed of the outcome of randomisation by the clinical trials' team, by telephone. BIS is then notified of the outcome of randomisation by the clinical trials' team (by secure email) in order that the service can book the first appointment with the patient in-line with the study protocols. The researcher is then notified that randomisation has occurred, and that the patient and service have been informed, but not the outcome of the randomisation. The purpose and need for single blinding is explained to patients and carers at the recruitment visit and they are reminded at the start of each subsequent blinded interview to try not to let the researcher know their group allocation. In addition, all data are handled using study identity numbers; group allocation identifiers will only be added at the analysis stage. A detailed description and discussion of single-blinding, piloted at Phase II, has been given elsewhere [20].

### Data collection interviews

Face to face mixed method interviews are conducted at a time and place convenient to the participant (usually their home). Careful attention is paid to both patient and carer need, in terms of fatigue. With permission, interviews are audio-recorded for transcription. Field notes are recorded after each interview. Interviews are conducted by one of three researchers: an experienced palliative care researcher (nursing background) or one of two sequentially employed researchers (nursing and psychology backgrounds respectively) who have been given full training (external in-depth interviewing course, internal training with the interview schedule and ongoing supervision). The researchers are allocated caseloads so that the same researcher conducts all interviews for patient X, their carer, their referrer and their non-referring health care professional.

### Measurement points

The data collection points (interviews) are outlined in Figures 1 and 2. Baseline interviews are collected for all respondents, regardless of diagnostic group, at t1, prior to randomisation.

For patients with non-malignant conditions and their carers, t2 occurs two weeks post t1 and the commencement of BIS (fast track group) or the entering of the waiting list (waiting list group), and t3 occurs four weeks post commencing BIS (fast track group) or the entering of the waiting list (waiting list group). The waiting list group have a t4 interview scheduled two weeks post their commencement of BIS (six weeks from t1). T5 occurs eight weeks after t1 (i.e. on discharge from BIS for the waiting list group or four weeks from discharge for fast track group). This model was successfully piloted at Phase II [20]. Therefore for those randomised to the fast track group, t2 and t3 represent mid-point and completion of the BIS intervention, for comparison with these same time-points in the contemporaneous control condition for those randomised to the waiting list group.

Due to the shorter time frame of the service model for patients with malignancies, there are no t2 or t4 interviews for patients or their carers in this group. Instead, t3 is conducted 2 weeks post t1 i.e. on discharge from BIS for the fast track group or just prior to commencement of BIS for the waiting list group. T5 then occurs four weeks after t1 (i.e. on discharge from BIS for the waiting list group or two weeks from discharge for fast track group). Therefore t3 is the time-point marking completion of the BIS intervention for those randomised to the fast track group, and the control condition in those randomised to the waiting list group.

### Sample size

Sample size was calculated using a power calculation, with the potential effect size and standard deviation informed by the existing literature and our Phase II trial [20]. The estimated standard deviation of the primary outcome measure is 2.5. In order to detect a 2-point difference in mean outcome between groups (equivalent to 0.8 sd effect size) with 80% power using a two-sided t-test at the 5% level of significance, it is necessary to analyse 26 patients per arm per disease group (trial) followed up with a primary outcome (total 104 patients). Therefore we propose to recruit a minimum of 120 patients, 60 per disease group, to ensure adequate power, effect size and allowance for attrition. Additionally, an adjustment for baseline in the analysis will improve the precision of the estimated intervention effect.

### Outcome measures

Table 3 outlines the baseline and outcome measures selected following a review of the literature and, to avoid duplication, a review of measures used by the clinical service [23-31]. Baseline characteristics and outcomes include patients' breathlessness, patient and carer distress due to breathlessness, patient and carer quality of life, patient and carer anxiety, other service use, caregiver burden, and patient and carer expectations of and satisfaction with BIS. The primary outcome measure is 'distress due to breathlessness' measured using a Numerical Rating Scale (NRS; 0-10).

**Table 3:** Patient and carer baseline and outcome measures for BIS Phase III RCT

Baseline characteristic/outcome	Instrument/measure
**PATIENT**	

Patient breathlessness	Numerical Rating Scale (NRS) for breathlessness [23] at worst/average over last 24 hours/now; modified Borg [24] at rest and on exertion & identification of activity that makes breathlessness worst

Patient distress due to breathlessness (primary outcome measure)	NRS for distress due to breathlessness (after Corner et al 1995 [25])

Patient quality of life	Chronic Respiratory Questionnaire [26] EQ-5D [27]

Patient anxiety & depression	Hospital Anxiety and Depression Scale [28]

Patient use of other services	CSRI [29]

Patient social functioning	No. of times patient goes out of the house (weekly average since last interview)

Patient experience of breathlessness & expectations of BIS/satisfaction & outcome of BIS	Brief qualitative interview: t1 - experience of breathlessness & expectations of BIS t3/t5 - satisfaction and outcome of BIS

**CARER**	

Carer's assessment of patient's breathlessness	Numerical Rating Scale (NRS) for breathlessness [23] at worst/average over last 24 hours/now; modified Borg [24] at rest and on exertion & identification of activity that makes breathlessness worst

Carer distress due to patient's breathlessness	NRS adapted for carer for distress to carer due to patient's breathlessness

Carer quality of life	EQ-5D [27]

Carer anxiety & depression	Hospital Anxiety and Depression Scale [28]

Carer's social functioning	No. of times carer goes out of the house (weekly average since last interview)

Caregiver burden	Burden Interview [30] Caregiver Appraisal Scale [31]

Carer experience of breathlessness & expectations of BIS/satisfaction & outcome of BIS	Brief qualitative interview: t1 - experience of breathlessness & expectations of BIS t3/t5 - satisfaction and outcome of BIS

At t1, prior to quantitative data collection, a brief qualitative interview is conducted to identify respondents' experiences of breathlessness, or caring for someone who is breathless, and their expectations of BIS. Following receipt of BIS (t3 for fast track group; t4 and t5 for waiting list group) and after quantitative data collection, a brief qualitative interview is conducted to identify respondents' experiences of and satisfaction with BIS. The resulting interview schedule is therefore both quantitative and qualitative at each measurement point except t2.

In addition, brief semi-structured interviews are conducted with patients' referrers two weeks after the referrer's first patient is discharged from BIS. This allows time for receipt of discharge documentation. Referrers are only approached for interview after their first referral to the service within the RCT; they are assured that they will not be asked for interview after every referral.

For patients with malignant conditions, a second health care professional interview is sought with a non-referring health care professional (identified by the clinical team) to capture potential knock-on effects (positive or negative) of a BIS referral on other health care professionals.

### Analysis

The same methods will be used to address the two research questions concerning the effectiveness of BIS and patient and carer distress and patient mastery of symptoms. Analyses will be presented separately for non-malignant and malignant participant groups. The baseline comparability of the arms will be examined by presenting pre-randomisation means and proportions of patient characteristics at timepoint t1 by randomised arm. The research questions will be addressed by cross-sectional comparison at t3 (end of intervention in fast track arm versus control condition in waiting list arm), using analysis of covariance adjusting for the baseline of the outcome within a linear regression framework. For the non-malignant group, this will be supported with the same analysis at t2.

Analyses will be primarily on an intention to treat basis. Secondary sensitivity analyses will be performed if there are deviations to the intervention protocol (requiring per protocol analysis) or there are baseline imbalances (requiring analysis adjustment). A graph will summarise the mean response over the time-points, with estimates and 95% confidence intervals for differences both between arms and within each arm over time from t3 to t5 (using paired t-tests). If a chi-squared test does not indicate that the t3 randomised effects are dissimilar in malignant and non-malignant groups, a simple average combined effect will be estimated with a narrower overall confidence interval to inform Phase IV. All analyses will be two-sided and assessed at the 5% significance level using SPSS software.

Qualitative data will be transcribed by an independent transcription company, checked and anonymised before analysis; transcripts will then be imported into NVivo software to facilitate the conduct of framework analysis [32]. Mixed method analyses will purposively sample qualitative data, based on quantitative findings.

### Cost-effectiveness

The cost of services used by each patient will be calculated from service activity data and appropriate unit costs. The BIS intervention costs will be based on staff salary costs, plus oncosts, overheads and equipment and divided by a relevant level of activity. Information on the use of other health and social services and informal care (proxy-valued as a homecare worker) will be collected with the Client Service Receipt Inventory (CSRI) [29]. This will be combined with appropriate unit cost data [e.g. [33]] to generate service costs. Cost comparisons will be made using bootstrapping methods to account for any skewness in data distribution. Cost-effectiveness will be assessed by combining cost data with that on the primary outcome measure (distress due to breathlessness) and quality-adjusted life years (QALYs), in the form of incremental cost-effectiveness ratios (if the intervention group has better outcomes and higher costs) and cost-effectiveness acceptability curves which will show the probability that the intervention is cost-effective for a range a values placed on a unit improvement in outcome compared to the waiting list control group.

### Monitoring

A Trial Management Group consisting of the clinical (BIS) and research teams (MF, JG/BBK) manage the everyday conduct of the trial through monthly face-to-face meetings and interim secure email contact. A Trial Advisory Group meets six monthly and consist of the clinical and research teams, the trial economist and statistician, academics (with expertise in complex interventions and palliative care RCTs), respiratory and oncology specialists, NHS managers, patients and carers.

### Approvals and trial registration

Ethical approval was obtained from the Cambridgeshire 2 Research Ethics Committee (08/H0308/157) and R&D approval and sponsorship from Addenbrooke's Hospital, Cambridge University Hospitals NHS Foundation Trust (A091322): current protocol version v5 110810. The trial is registered with http://ClinicalTrials.gov (NCT00678405) and Current Controlled Trials (ISRCTN04119516).

## Discussion

This is the first evaluation of an intervention for breathlessness in advanced disease to have followed the MRC framework; only recently has the framework been used in a palliative care setting [34]. It is also one of the very few trials in palliative care to propose the use of fast track methodology [20,34,35], and the first, as far as we are aware, to combine this with single-blinding (other than our Phase II trial [20]).

The Phase III protocol described here was developed based on our Phase II pilot trial [20]. One limitation of this is that the pilot focused only on patients with COPD. The reason for this was that COPD patients made (and continue to make) up the largest group of patients referred to BIS and existing studies of breathlessness interventions had focused on patients with malignancies. Funding was sought for a Phase II trial with patients with malignancies but was unsuccessful. Thus the methods outlined here for Phase III have not been tested on patients with malignant conditions.

A further limitation of this Phase III study may be that although some outcome measures were revised as a result of Phase II, the new measures adopted for Phase III were not subjected to formal piloting, nor were the economic evaluation measures i.e. EQ-5D and CSRI.

The MRC framework has provided a useful structure for the systematic development and evaluation of BIS [3,10,20,21], however, as Booth [36] has commented, the length of time for completion of the framework is considerable. The development work for the Pre-Clinical Phase began in 1998 and Phase III is due for completion in 2012. In this time there will have been changing contextual factors that will need to be considered and accounted for in terms of their impact on both the development of the service and the outcome of its evaluation. Campbell et al (2007) noted that the setting in which an intervention is given must be understood before the intervention can be used elsewhere [37]; this will be particularly important as the evaluation of BIS moves into Phase IV.

The results of this Phase III trial will therefore provide evidence of the effectiveness and cost-effectiveness of BIS, inform the long term development of the service and its implementation in other centres nationally and internationally, as well as adding to methodological developments in palliative care research and the development and evaluation of complex interventions.

## List of abbreviations

BIS: Breathlessness Intervention Service; RCT: randomised controlled trial; COPD: chronic obstructive pulmonary disease; MRC: Medical Research Council; NRS: numerical rating scale; CSRI: Client Services Receipt Inventory.

## Competing interests

MF, TP, PM, IJH, JG, and BBK declare that they have no competing interests. SB (founder of service) is one of the clinicians providing the intervention (BIS).

## Authors' contributions

MF co-designed Phase III, co-applied for Phase III funding, gained LREC and R&D approval, is main researcher on Phase III, and authored the background document to and subsequent drafts of this paper. SB founded service and evaluation, designed the original intervention and developed it subsequently with other BIS clinicians. SB is also an intervention provider (clinician), co-designed Phase III, co-applied for Phase III funding, and contributed to revising the paper. IJH co-designed Phase III, co-applied for funding, and contributed to revising the paper. TP co-designed Phase III (statistics), co-applied for funding, developed the randomisation sequence, and contributed to revising the paper. PM co-designed Phase III (economic evaluation), co-applied for funding, and contributed to revising the paper. JG was the research associate during the first half of the Phase III RCT and led the initial drafting of this paper working closely with MF. BBK is the research associate for the second half of the Phase III RCT, has contributed to protocol revisions and contributed to revising the paper. All authors read and approved the final draft.

## References

[B1] Higginson I McCarthy M Measuring symptoms in terminal cancer: are pain and dyspnoea controlled? J R Soc Med 1989 82 264 7 2474072 10.1177/014107688908200507 PMC1292128

[B2] Skevington M Pilaar M Routh D On the language of breathlessness Psychol Health 1997 12 677 89 10.1080/08870449708407414

[B3] Booth S Silvester S Todd C Breathlessness in cancer and chronic obstructive pulmonary disease: using a qualitative approach to describe the experience of patients and carers Palliat Support Care 2003 1 4 337 44 16594223 10.1017/s1478951503030499

[B4] Dudgeon D Christiansen L Sloan J Lertzman M Clement K Dyspnoea in cancer patients: prevalence and associated factors J Pain Symptom Manage 2001 21 2 95 102 10.1016/S0885-3924(00)00258-X 11226761

[B5] Muers MF Round CE Palliation of symptoms in non-small cell lung cancer: a study by the Yorkshire Regional Cancer Organisation Thoracic Group Thorax 1993 48 339 43 10.1136/thx.48.4.339 7685550 PMC464429

[B6] Health and Safety Laboratory Projection of mesothelioma mortality in Great Britain (RR 728) 2009 London: Health and Safety Executive

[B7] Edmonds P Karlsen S A comparison of the palliative care needs of patients dying from chronic respiratory diseases and lung cancer Palliat Med 2001 15 287 95 10.1191/026921601678320278 12054146

[B8] Hopwood P Stephens R Symptoms at presentation for treatment in patients with lung cancer. Implications for the evaluation of palliative treatment Br J Cancer 1995 71 633 6 10.1038/bjc.1995.124 7533520 PMC2033650

[B9] Booth S Moosavi SH Higginson IJ The etiology and management of intractable breathlessness in patients with advanced cancer: a systematic review of pharmacological therapy Nat Clin Pract Oncol 2008 5 2 90 100 10.1038/ncponc1034 18235441

[B10] Booth S Farquhar M Gysels M Bausewein C Higginson IJ The impact of a breathlessness intervention service (BIS) on the lives of patients with intractable dyspnoea: a qualitative Phase I study Palliat Support Care 2006 4 287 93 17066970 10.1017/s1478951506060366

[B11] Seamark DA Blake SD Seamark CJ Halpin DM Living with severe chronic obstructive pulmonary disease (COPD): perceptions of patients and their carers. An interpretative phenomenological analysis Palliat Med 2004 18 7 619 25 10.1191/0269216304pm928oa 15540670

[B12] NCCCC National guidelines on management of chronic obstructive pulmonary disease in adults in primary and secondary care Thorax 2004 59 Suppl 1 1 232 15041752 PMC1766028

[B13] Goldberg R Hillberg R Reinecker L Goldstein R Evaluation of patients with severe pulmonary disease before and after pulmonary rehabilitation Disabil Rehabil 2004 26 11 641 8 10.1080/09638280410001663120 15204502

[B14] Man WDC Polkey MI Donaldson N Gray BJ Moxham J Community pulmonary rehabilitation after hospitalisation for acute exacerbations of chronic obstructive pulmonary disease: randomised controlled study BMJ 2004 329 1209 13 10.1136/bmj.38258.662720.3A 15504763 PMC529363

[B15] Corner J Plant H A'Hern R Non-pharmacological intervention for breathlessness in lung cancer Palliat Med 1996 10 299 305 10.1177/026921639601000405 8931065

[B16] Bredin M Corner J Krishnasamy M Plant H Bailey C A'Hern R Multicentre randomised control trial of a nursing intervention for breathlessness in patients with lung cancer BMJ 1999 318 901 4 10102851 10.1136/bmj.318.7188.901 PMC27809

[B17] Hately J Laurence V Scott A Breathlessness clinics within specialist palliative care settings can improve the quality of life and functional capacity of patients with lung cancer Palliat Med 2003 17 410 7 10.1191/0269216303pm752oa 12882259

[B18] Booth S Moffat C Farquhar M Higginson IJ Bausewein C Burkin J Developing a breathlessness service for patients with palliative and supportive care needs, irrespective of diagnosis J Palliat Care 2011 in press 21510129

[B19] MRC A framework for development and evaluation of RCTs for complex interventions to improve health 2000 London: MRC

[B20] Farquhar M Higginson IJ Fagan P Booth S The feasibility of a single-blinded fast-track pragmatic randomised controlled trial of a complex intervention for breathlessness in advanced disease BMC Palliat Care 2009 8 9 10.1186/1472-684X-8-9 19583857 PMC2731082

[B21] Farquhar M Higginson IJ Fagan P Booth S Results of a pilot investigation into a complex intervention for breathlessness in advanced chronic obstructive pulmonary disease (COPD): brief report Palliat Support Care 2010 8 2 143 9 10.1017/S1478951509990897 20307365

[B22] Terry W Olson LG Ravenscroft P Wilss L Boulton-Lewis G Hospice patients' views on research in palliative care Intern Med J 2006 36 406 13 10.1111/j.1445-5994.2006.01078.x 16780445

[B23] Gift AG Narsavage G Validity of the numeric rating scale as a measure of dyspnea Am J Crit Care 1998 7 3 200 4 9579246

[B24] Burdon JGW Juniper EF Killian FE Hargreave FE Campbell EJM The perception of breathlessness in asthma Am Rev Respir Dis 1982 126 825 828 7149447 10.1164/arrd.1982.126.5.825

[B25] Corner J Plant H Warner L Developing a nursing approach to managing dyspnoea in lung cancer Int J Palliat Nurs 1995 1 5 11 10.12968/ijpn.1995.1.1.5 29323561

[B26] Guyatt GH Berman LB Townsend M Pugsley SO Chambers LW A measure of quality of life for clinical trials in chronic lung disease Thorax 1987 42 773 8 10.1136/thx.42.10.773 3321537 PMC460950

[B27] EuroQoL Group EuroQoL-a new facility for the measurement of health-related quality of life Health Policy 1990 16 199 208 10109801 10.1016/0168-8510(90)90421-9

[B28] Zigmond AS Snaith RP The Hospital Anxiety and Depression Scale Acta Psychiatr Scand 1983 67 361 70 10.1111/j.1600-0447.1983.tb09716.x 6880820

[B29] Beecham J Knapp M Thornicroft G Costing psychiatric interventions Measuring Mental Health Needs 2001 2 London: The Royal College of Psychiatrists 200 24

[B30] Zarit SH Reever KE Bach-Peterson J Relatives of the impaired elderly: correlates of feelings of burden Gerontologist 1980 20 6 649 55 7203086 10.1093/geront/20.6.649

[B31] Lawton MP Kleban MH Moss M Rovine M Glicksman A Measuring caregiving appraisal J Gerontol 1989 44 3 61 71 10.1093/geronj/44.3.p61 2715587

[B32] Ritchie J Spencer L Bryman A, Burgess RG Qualitative data analysis for applied policy research Analyzing Qualitative Data 1994 London: Routledge 173 94

[B33] Curtis L Netten A Unit costs of health and social care 2006 Canterbury: PSSRU

[B34] Higginson IJ Vivat B Silber E Saleem T Burman R Hart S Edmonds P Study protocol: delayed intervention randomised controlled trial within the Medical Research Council (MRC) Framework to assess the efffectiveness of a new palliative care service BMC Palliat Care 2006 5 7 10.1186/1472-684X-5-7 17014714 PMC1615868

[B35] McWhinney IR Bass MJ Donner D Evaluation of a palliative care service: problems and pitfalls BMJ 1994 309 1340 2 7532501 10.1136/bmj.309.6965.1340 PMC2541867

[B36] Booth S Improving the Palliative Care of Patients with Intractable Breathlessness MD thesis 2008 University of London

[B37] Campbell NC Murray E Darbyshire J Emery J Farmer A Griffiths F Guthrie B Lester H Wilson P Kinmonth AL Designing and evaluating complex interventions to improve health care BMJ 2007 334 7591 455 9 10.1136/bmj.39108.379965.BE 17332585 PMC1808182

